# Supplementary material for: The Visual Patient Avatar ICU Facilitates Information Transfer of Written Information by Visualization: A Multicenter Comparative Eye-Tracking Study
Source: Diagnostics (Basel). 2023 Nov 12;13(22):3432. doi: 10.3390/diagnostics13223432 (PMC10670428; doi:10.3390/diagnostics13223432)
Supplement: Supplementary file 1 [file diagnostics-13-03432-s001.zip › diagnostics-2604662-supplementary/Additional Files/AddFile2_allscenarios.pdf]

Visual Patient
Intensive Care

Computer-based multicenter study

Instructions

- Thank you very much for participating in this study!

- Main topic of our study group: new forms of presentation of medical parameters

Aim

  - Improving their perception
  - Improving situational awareness for possible changes

- Technique for vital sign presentation: “Visual Patient"
  - animated virtual model of the monitored patient

Instructions

- Study: Comparison of two ways of displaying vital signs
  “Visual patient” vs. “conventional monitor”
- 1. Teachingvideo for the «Visual Patient»
- 2. Example of a «conventional monitor»
- 3. One by one: 10 monitor slides (15 seconds each)
  - 5x conventional design
  - 5x “Visual Patient"
- Try to remember as many vital signs and installations as possible
- Please answer the questions on the Ipad after each example

Instructions

Table with the defined limit values

Defined limits of vital signs

Parameter	Low VP	High VP	(Units)
HR (ECG)	<55	>100	/min
Pulse	<55	>100	/min
ABP (Mean)	<65	>100	mmHg
NBP (Mean)	<65	>100	mmHg
CVP (Mean)	<4	>12	mmHg
STE	<-0.2	>0.2	mV
SpO2	<94	none	%
RR	<8	>16	/min
TV	<350	>750	ml
etCO2	<35	>45	mmHg
Tcore	<36.0	≥37.5	°C
BIS	≤60	>60	(-)
TOF rat.	<90	≥90	%
CI	<2.5	>4	l/m^2/min
Peak airway pressure (PIP)	<10	>30	mbar
Insp. O2	<40	≥80	%

Introduction

- Teachingvideo «Visual patient»

Introduction

- Example «conventional monitor»

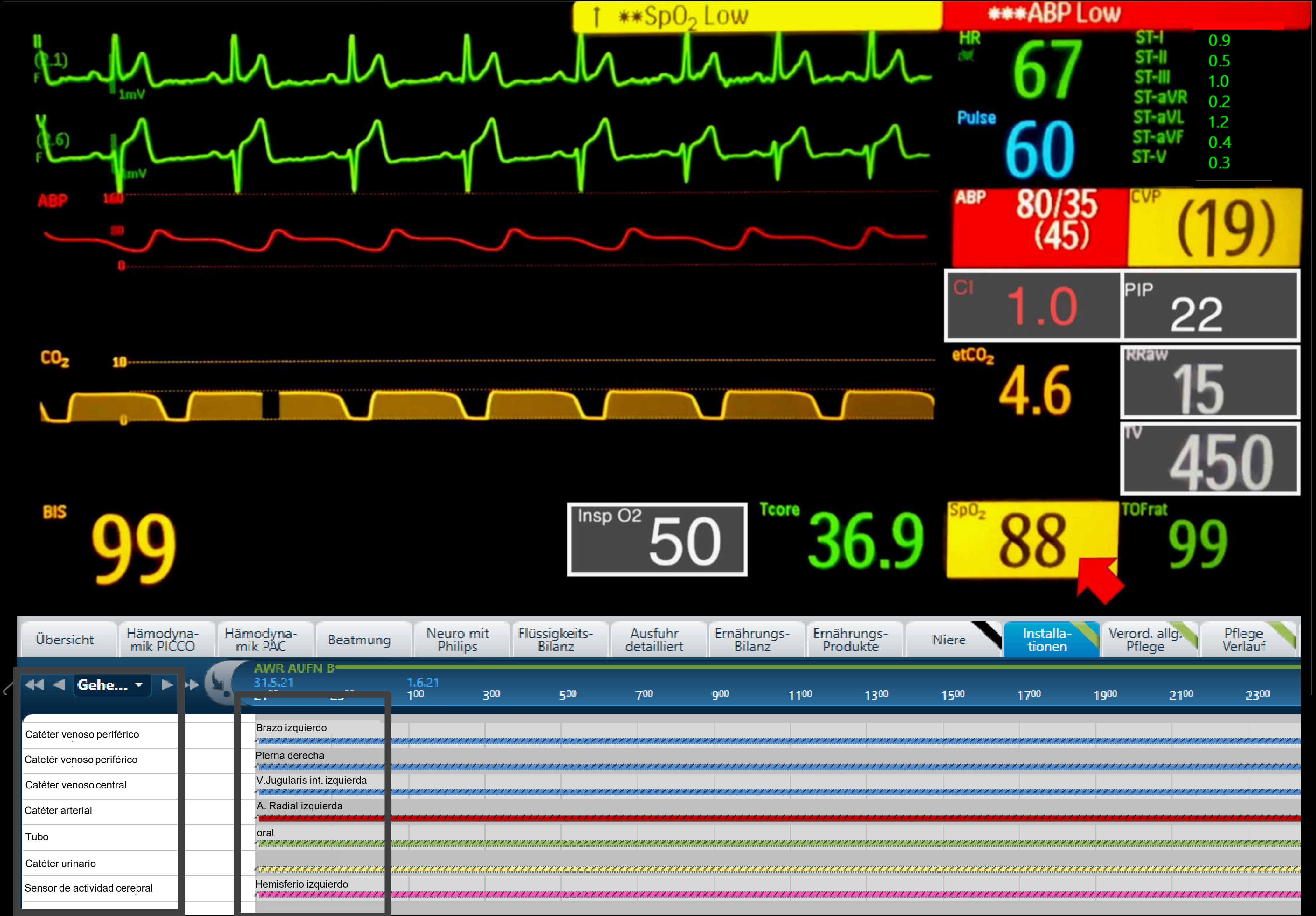
**SpO2 Low
***ABP Low
HR
67
Pulse
60
ST-I 0.9
ST-II 0.5
ST-III 1.0
ST-aVR 0.2
ST-aVL 1.2
ST-aVF 0.4
ST-V 0.3
ABP
80/35
(45)
CVP
(19)
CI
1.0
PIP
22
CO2
etCO2
4.6
RRaw
15
TV
450
BIS
99
Insp O2
50
Tcore
36.9
SpO2
88
TOFrat
99
Übersicht
Hämodyna-mik PICCO
Hämodyna-mik PAC
Beatmung
Neuro mit Philips
Flüssigkeits-Bilanz
Ausfuhr detailliert
Ernährungs-Bilanz
Ernährungs-Produkte
Niere
Installa-tionen
Verord. allg. Pflege
Pflege Verlauf
Gehe...
AWR AUFN B
31.5.21
1.6.21
Catéter venoso periférico
Catetér venoso periférico
Catéter venoso central
Catéter arterial
Tubo
Catéter urinario
Sensor de actividad cerebral
Brazo izquierdo
Pierna derecha
V.Jugularis int. izquierda
A. Radial izquierda
oral
Hemisferio izquierdo

Survey

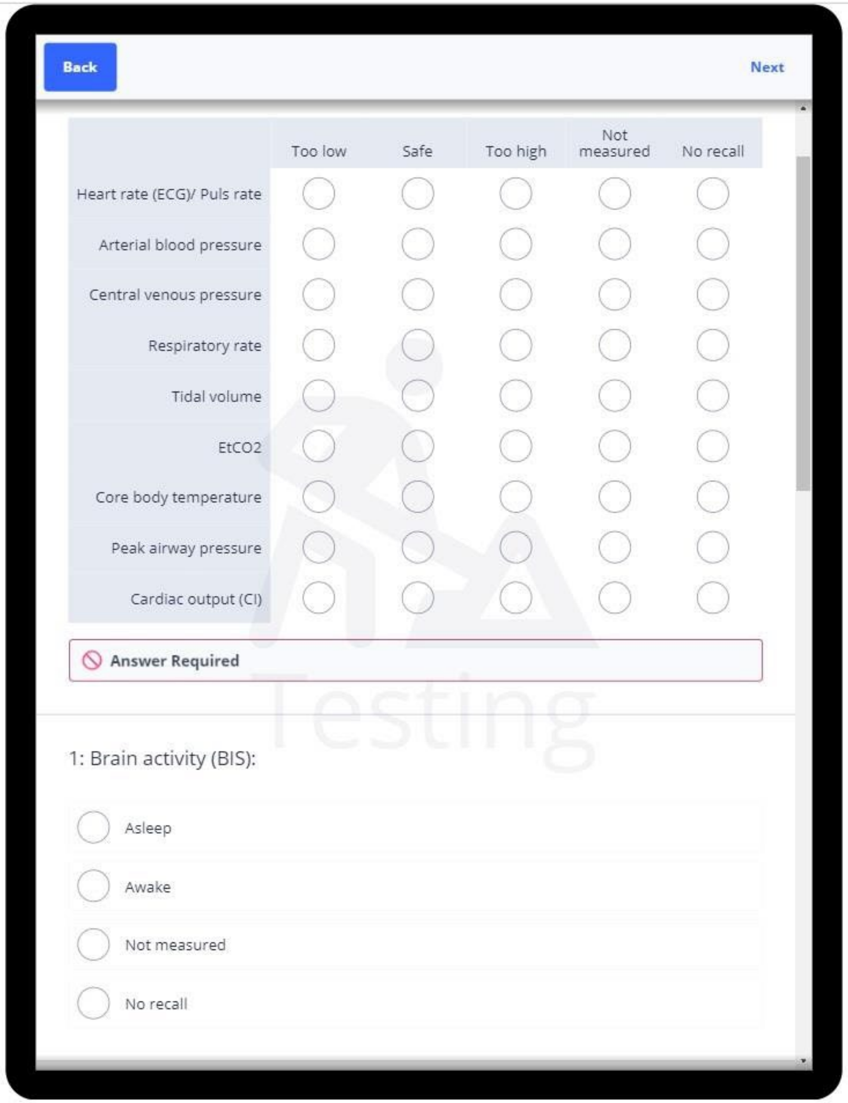
Back
Next
Too low
Safe
Too high
Not measured
No recall
Heart rate (ECG)/ Puls rate
Arterial blood pressure
Central venous pressure
Respiratory rate
Tidal volume
EtCO2
Core body temperature
Peak airway pressure
Cardiac output (CI)
Answer Required
Testing
1: Brain activity (BIS):
Asleep
Awake
Not measured
No recall

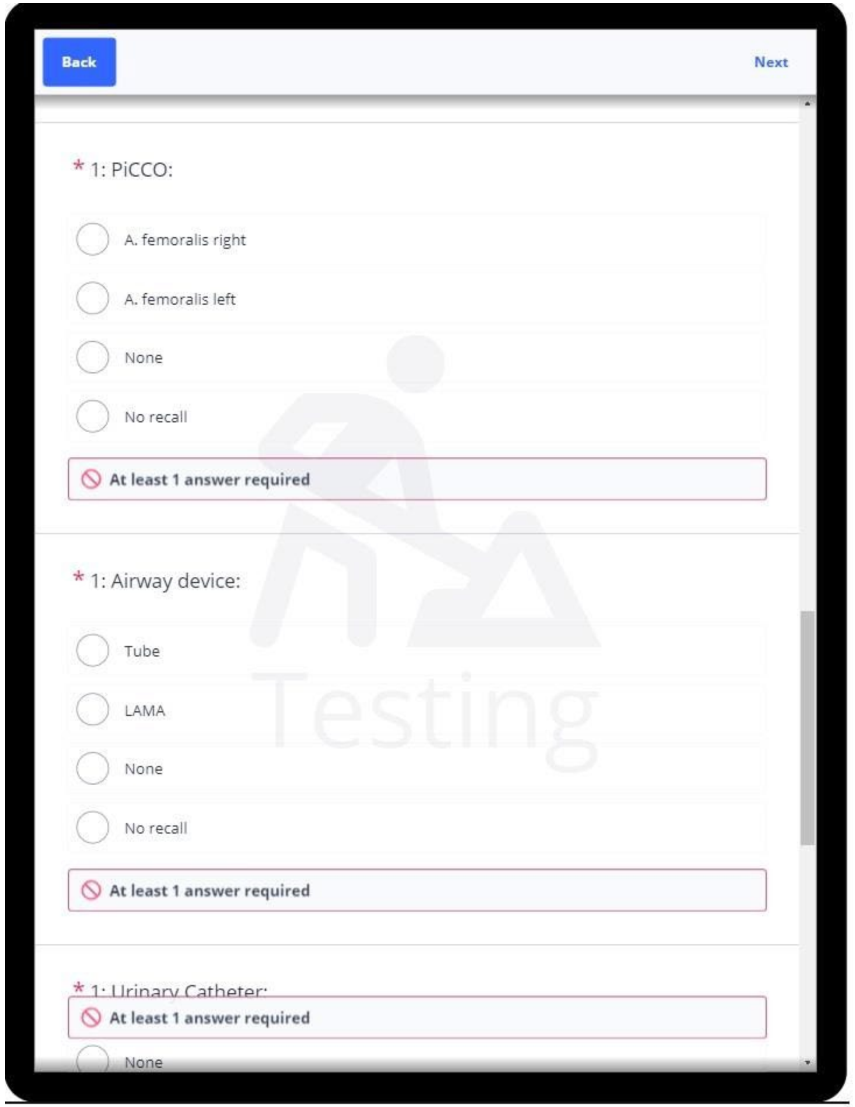
Back
Next
* 1: PiCCO:
A. femoralis right
A. femoralis left
None
No recall
At least 1 answer required
* 1: Airway device:
Tube
LAMA
None
No recall
At least 1 answer required
Testing
* 1: Urinary Catheter:
At least 1 answer required
None

Survey

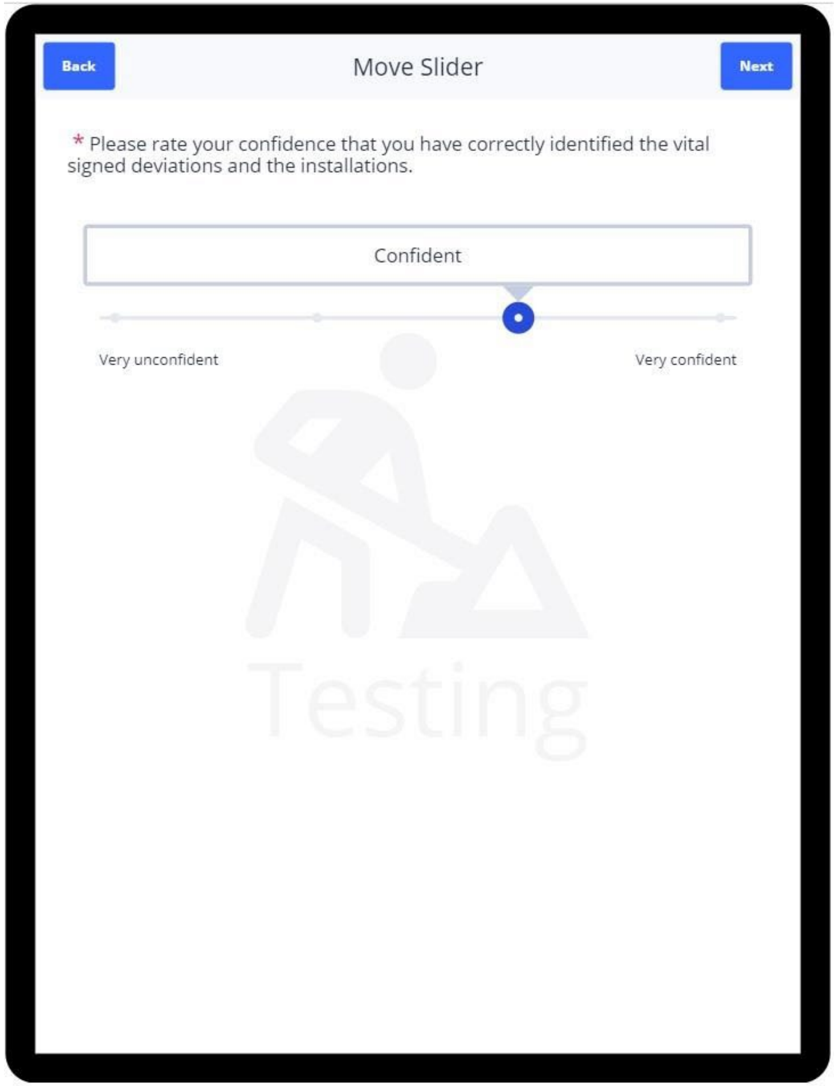
Back
Move Slider
Next
* Please rate your confidence that you have correctly identified the vital signed deviations and the installations.
Confident
Very unconfident
Very confident
Testing

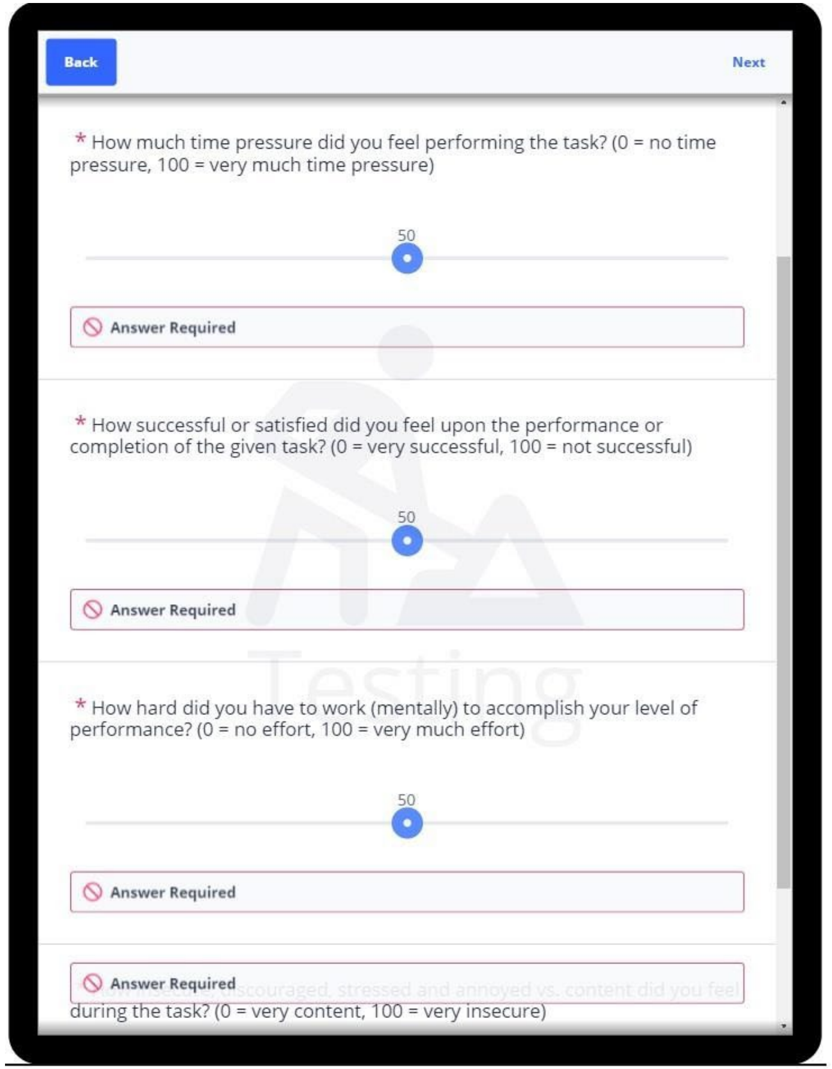
Back
Next
* How much time pressure did you feel performing the task? (0 = no time pressure, 100 = very much time pressure)
50
Answer Required
* How successful or satisfied did you feel upon the performance or completion of the given task? (0 = very successful, 100 = not successful)
50
Answer Required
* How hard did you have to work (mentally) to accomplish your level of performance? (0 = no effort, 100 = very much effort)
50
Answer Required
Answer Required
during the task? (0 = very content, 100 = very insecure)

Case 1

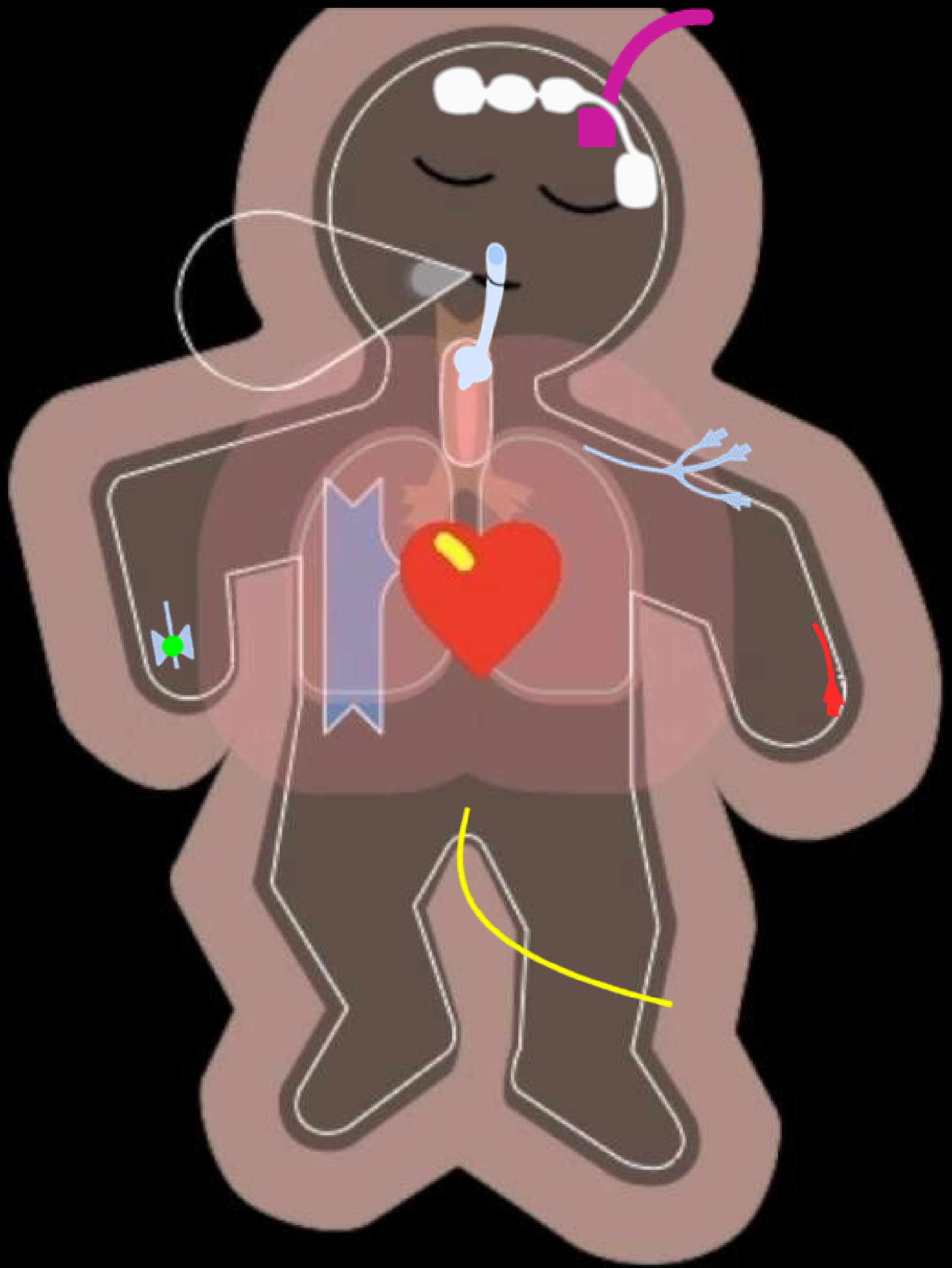

VP1

Case 2

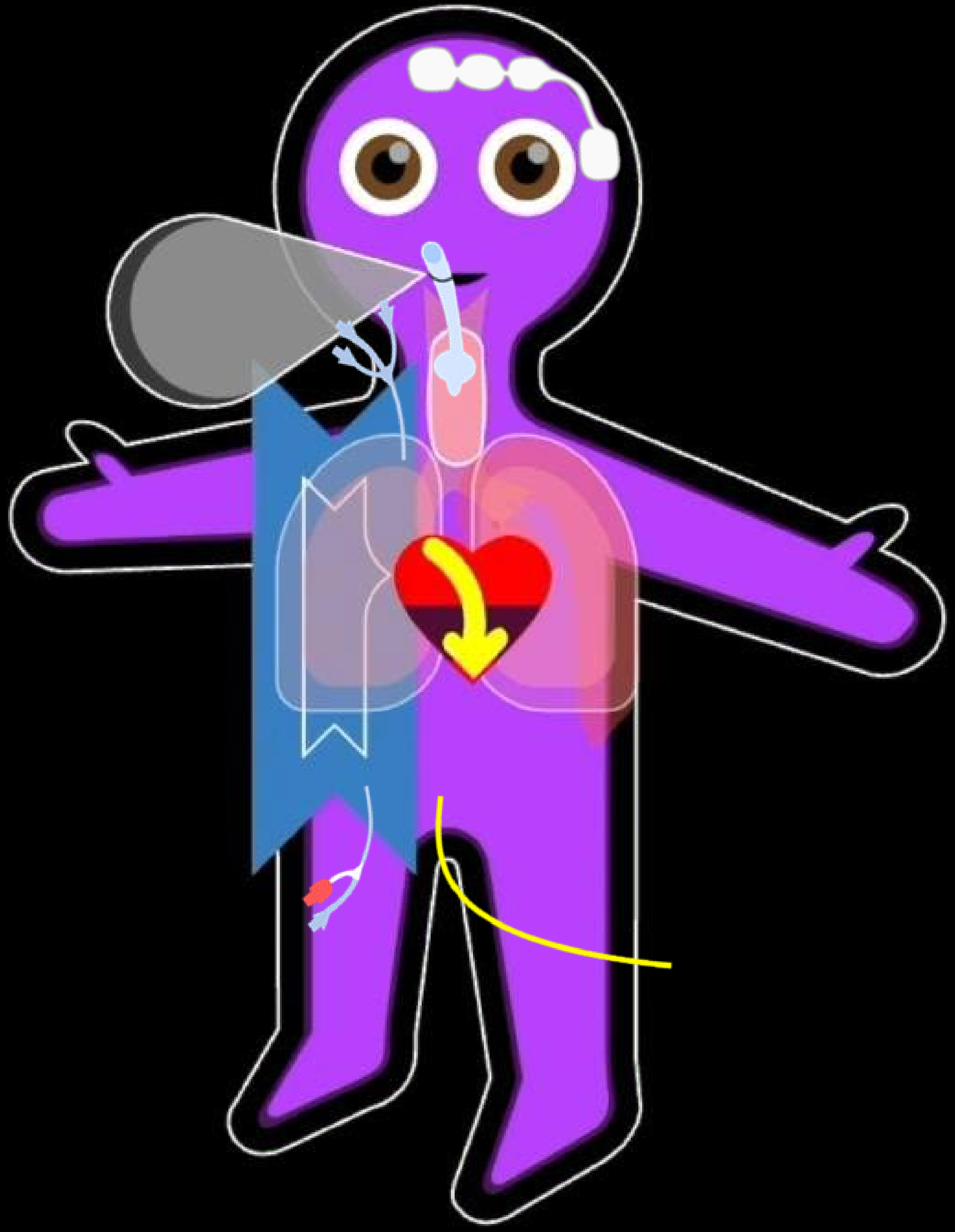

Case 3

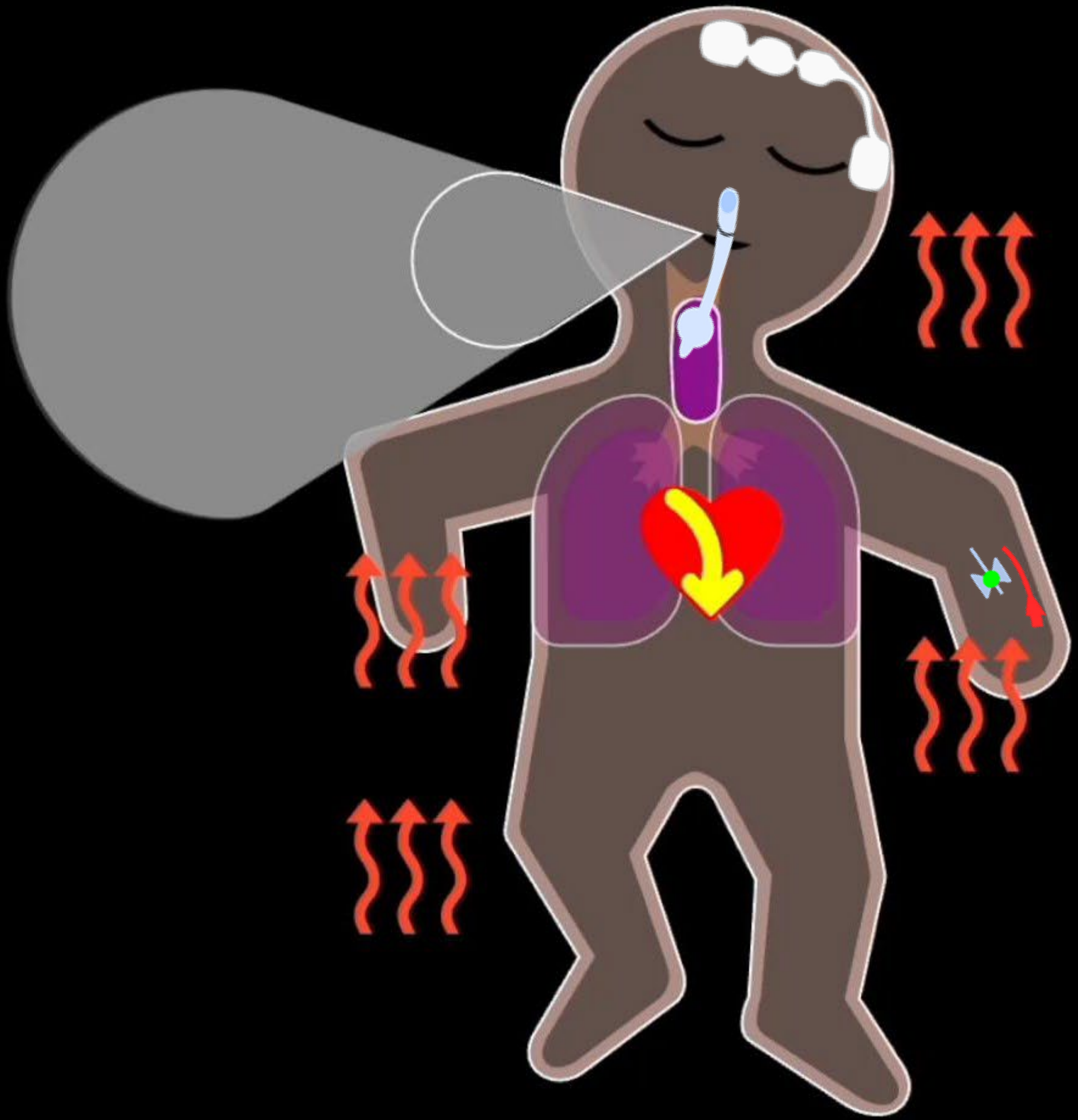

VP 3

Case 4

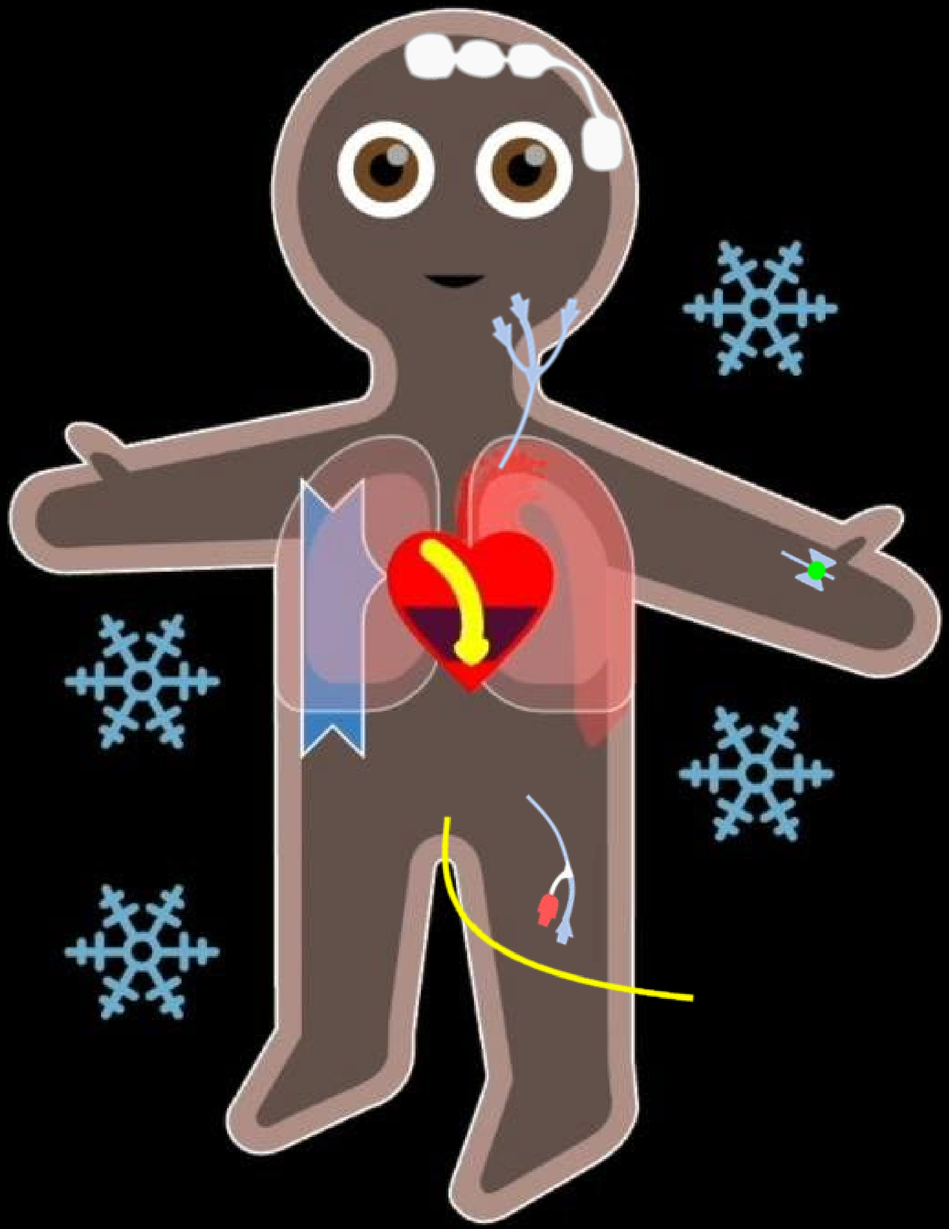

Case 5

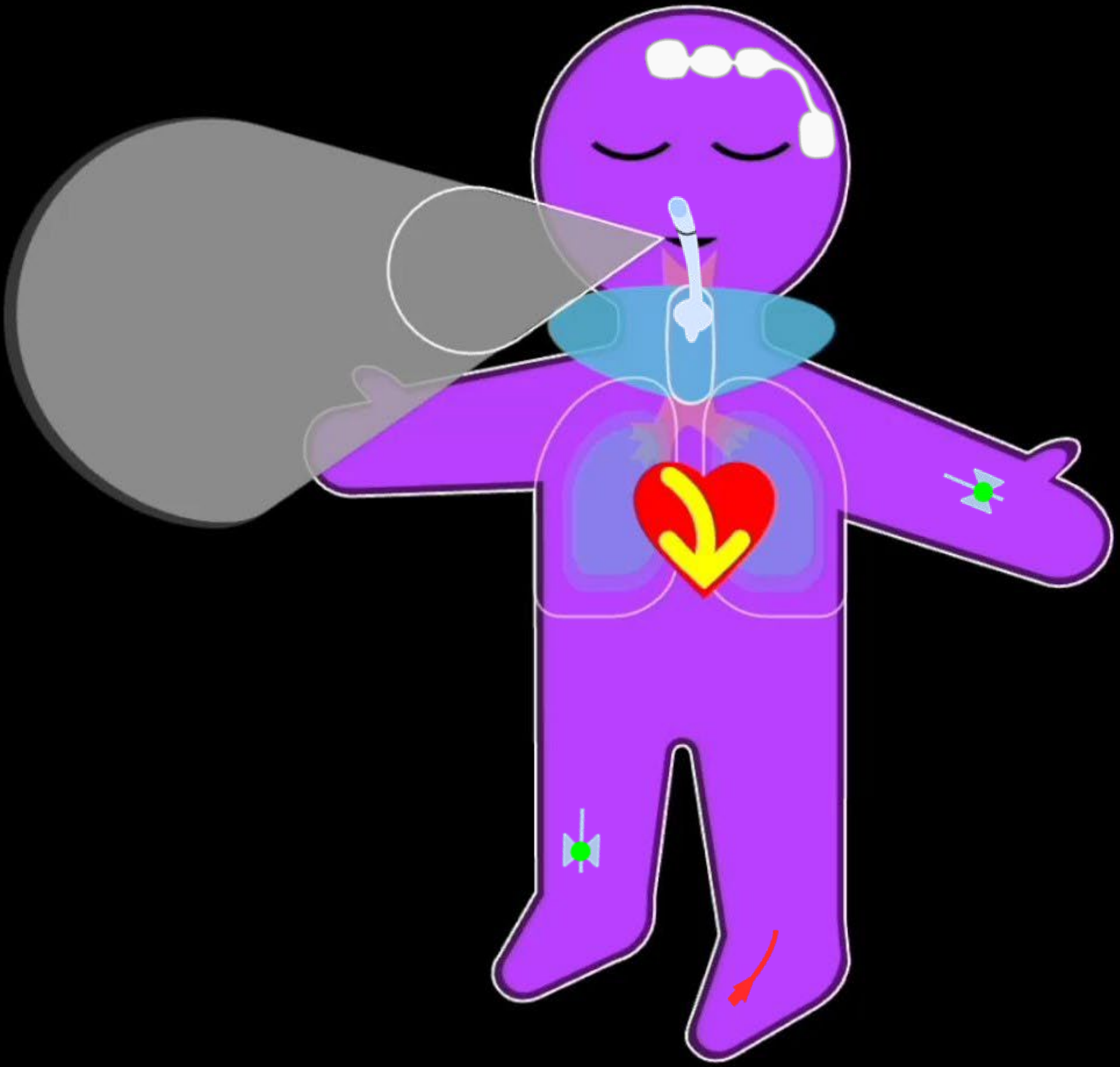

Case 6

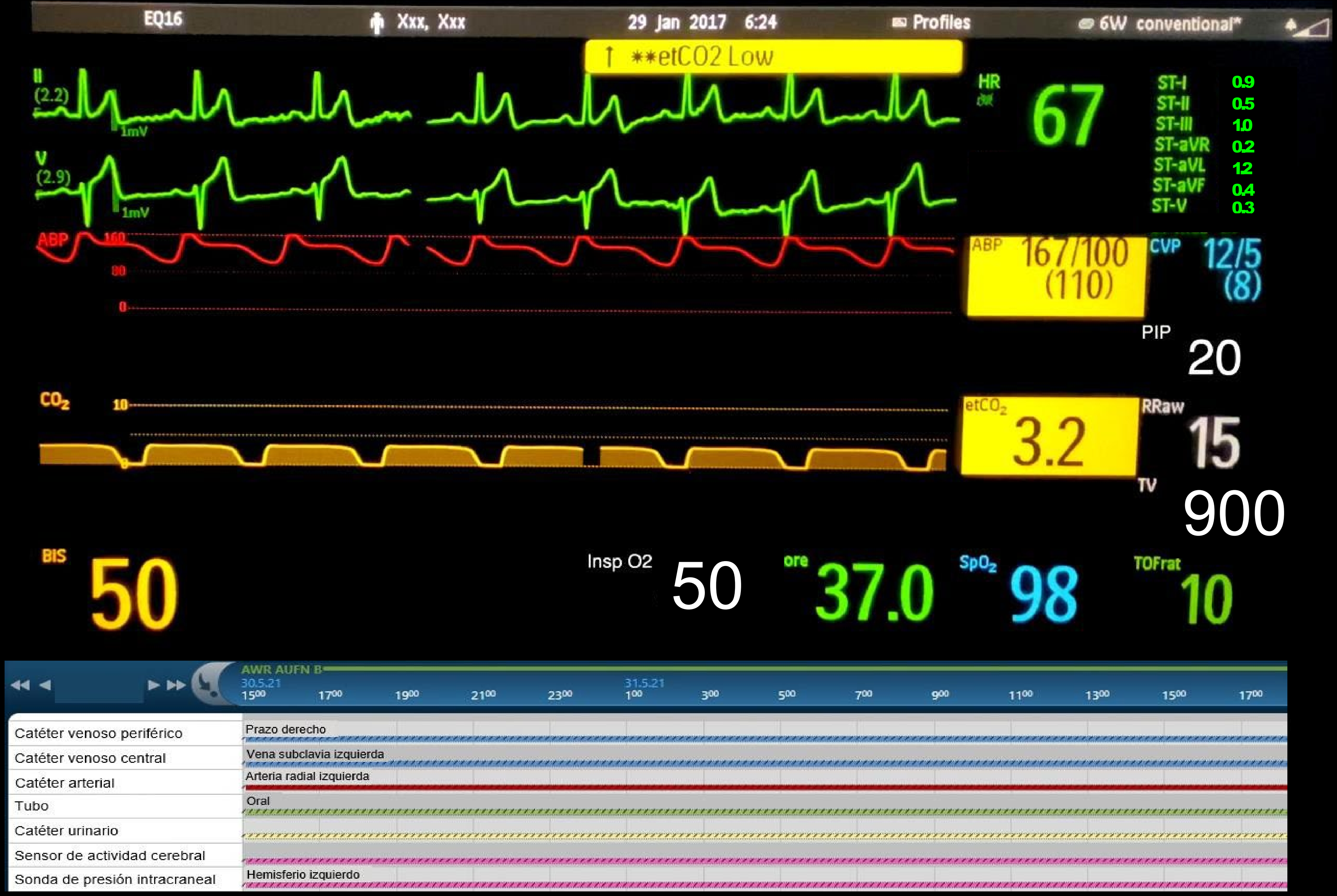
EQ16
Xxx, Xxx
29 Jan 2017 6:24
Profiles
6W conventional*
**etCO2 Low
II (2.2)
V (2.9)
1mV
ABP
160
80
0
HR
67
ST-I 0.9
ST-II 0.5
ST-III 1.0
ST-aVR 0.2
ST-aVL 1.2
ST-aVF 0.4
ST-V 0.3
ABP 167/100 (110)
CVP 12/5 (8)
PIP 20
CO_2
10
$etCO_2$ 3.2
RRaw 15
TV 900
BIS 50
Insp O2 50
ore 37.0
SpO_2 98
TOFrat 10
AWR AUFN B
30.5.21
31.5.21
15 00 17 00 19 00 21 00 23 00 1 00 3 00 5 00 7 00 9 00 11 00 13 00 15 00 17 00
Catéter venoso periférico
Prazo derecho
Catéter venoso central
Vena subclavia izquierda
Catéter arterial
Arteria radial izquierda
Tubo
Oral
Catéter urinario
Sensor de actividad cerebral
Sonda de presión intracraneal
Hemisferio izquierdo

Case 7

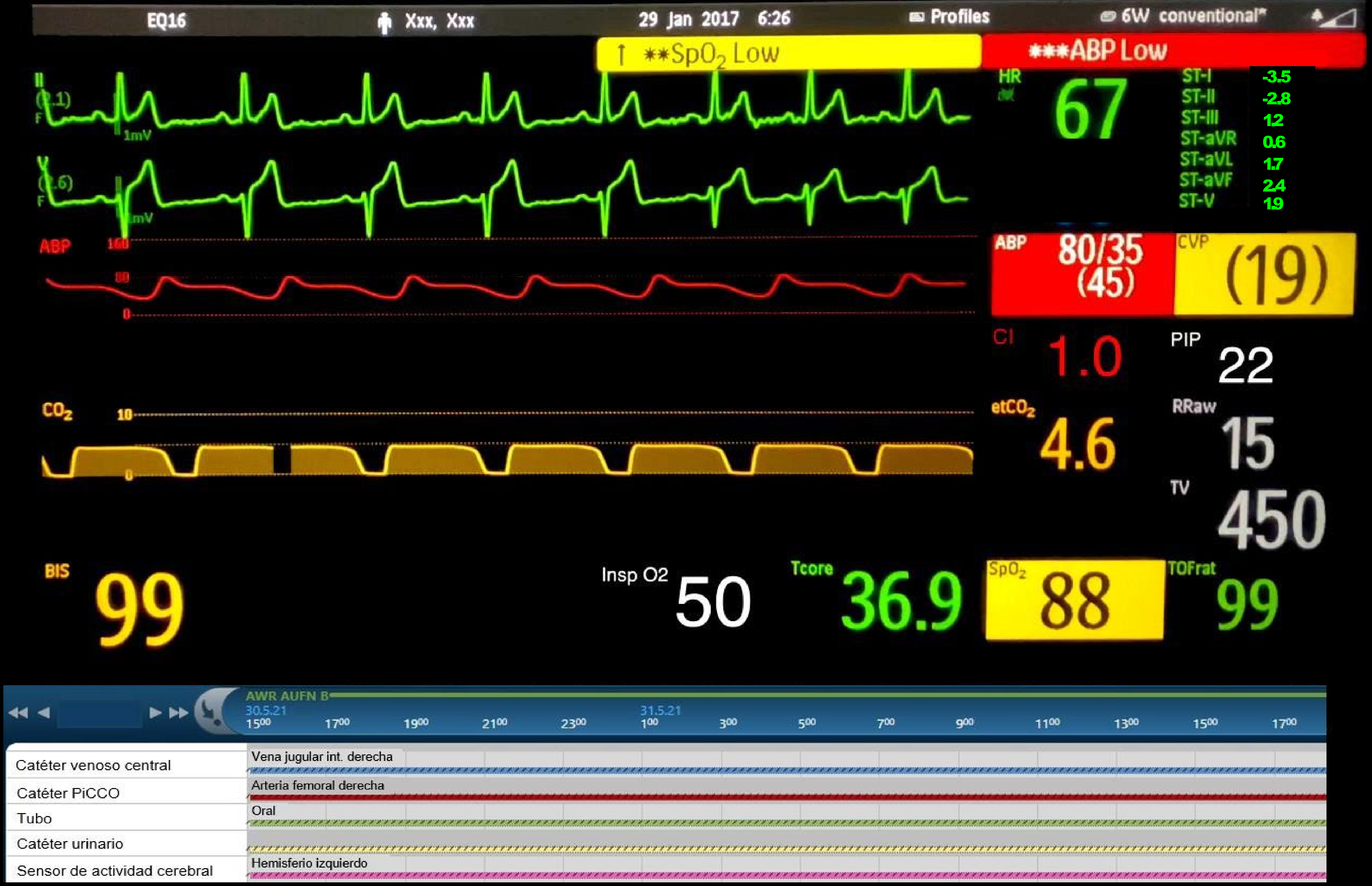
EQ16
Xxx, Xxx
29 Jan 2017 6:26
Profiles
6W conventional*
↑ **SpO2 Low
***ABP Low
II
1mV
V
1mV
ABP
160
80
0
HR
67
ST-I -3.5
ST-II -2.8
ST-III 1.2
ST-aVR 0.6
ST-aVL 1.7
ST-aVF 2.4
ST-V 1.9
ABP 80/35 (45)
CVP (19)
CI 1.0
PIP 22
CO2
10
0
etCO2 4.6
RRaw 15
TV 450
BIS 99
Insp O2 50
Tcore 36.9
SpO2 88
TOFrat 99
AWR AUFN B
30.5.21
31.5.21
1500 1700 1900 2100 2300 100 300 500 700 900 1100 1300 1500 1700
Catéter venoso central
Vena jugular int. derecha
Catéter PiCCO
Arteria femoral derecha
Tubo
Oral
Catéter urinario
Sensor de actividad cerebral
Hemisferio izquierdo

C 2

Case 8

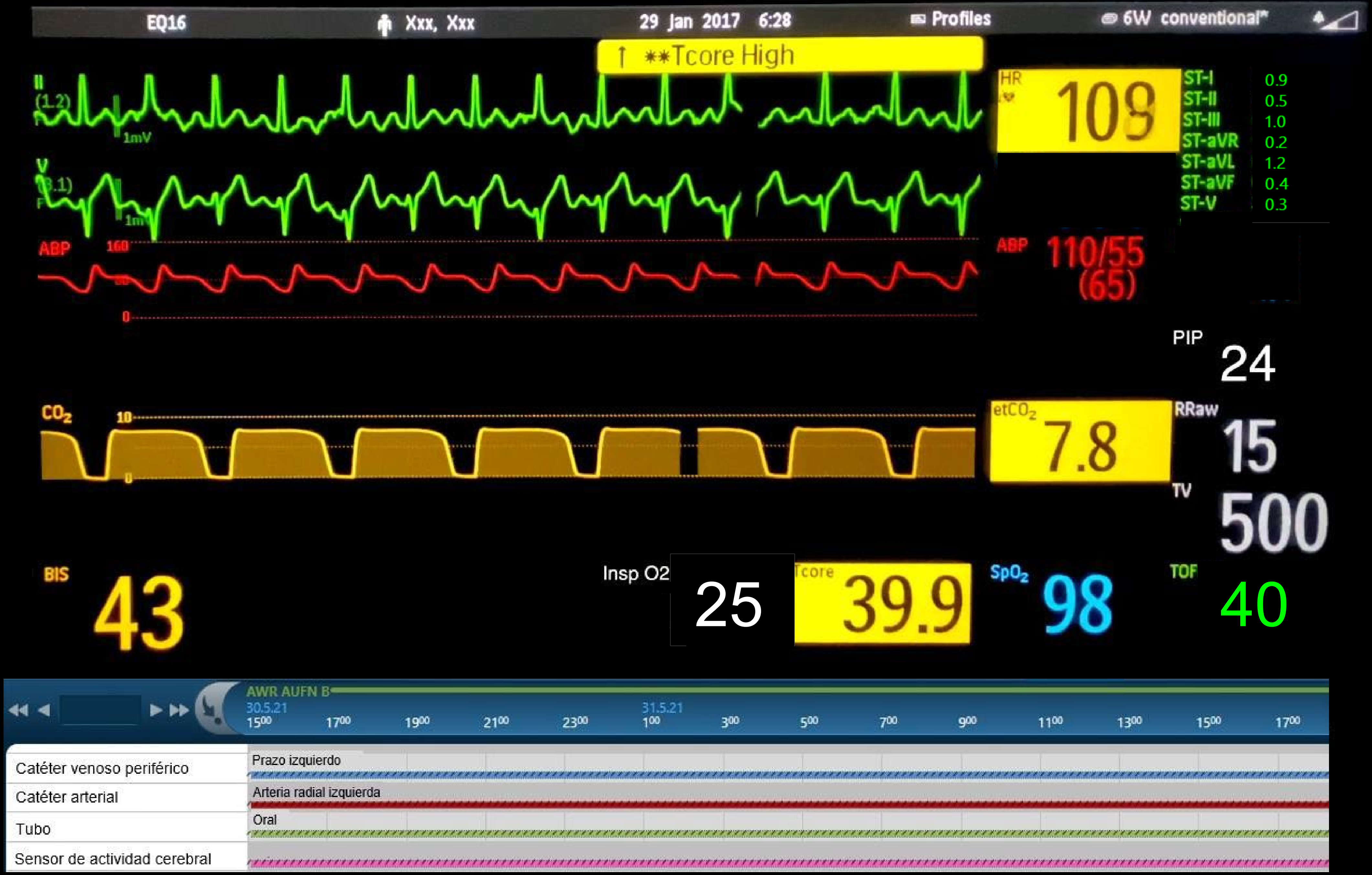
EQ16
Xxx, Xxx
29 Jan 2017 6:28
Profiles
6W conventional*
**Tcore High
HR
108
ST-I 0.9
ST-II 0.5
ST-III 1.0
ST-aVR 0.2
ST-aVL 1.2
ST-aVF 0.4
ST-V 0.3
ABP
160
0
ABP 110/55 (65)
PIP
24
CO2
10
etCO2
7.8
RRaw
15
TV
500
BIS
43
Insp O2
25
Tcore
39.9
SpO2
98
TOF
40
AWR AUFN B
30.5.21
31.5.21
Catéter venoso periférico
Prazo izquierdo
Catéter arterial
Arteria radial izquierda
Tubo
Oral
Sensor de actividad cerebral

C 3

Case 9

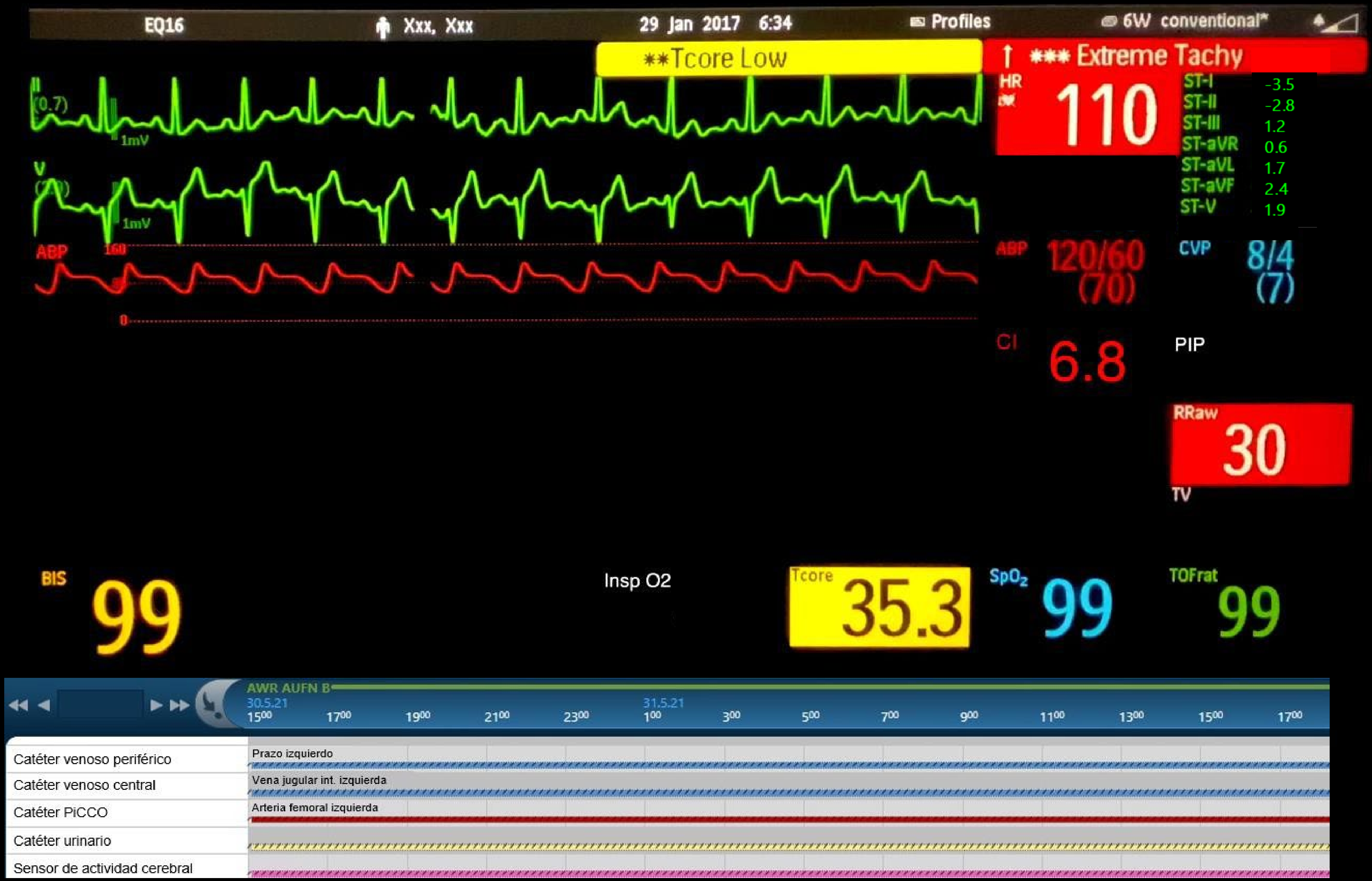
EQ16
Xxx, Xxx
29 Jan 2017 6:34
Profiles
6W conventional*
**Tcore Low
*** Extreme Tachy
HR
110
ST-I -3.5
ST-II -2.8
ST-III 1.2
ST-aVR 0.6
ST-aVL 1.7
ST-aVF 2.4
ST-V 1.9
II
(0.7)
1mV
V
1mV
ABP
160
0
ABP 120/60 (70)
CVP 8/4 (7)
CI 6.8
PIP
RRaw 30
TV
BIS 99
Insp O2
Tcore 35.3
SpO2 99
TOFrat 99
AWR AUFN B
30.5.21
31.5.21
15:00 17:00 19:00 21:00 23:00 1:00 3:00 5:00 7:00 9:00 11:00 13:00 15:00 17:00
Catéter venoso periférico
Prazo izquierdo
Catéter venoso central
Vena jugular int. izquierda
Catéter PiCCO
Arteria femoral izquierda
Catéter urinario
Sensor de actividad cerebral

C 4

Case 10

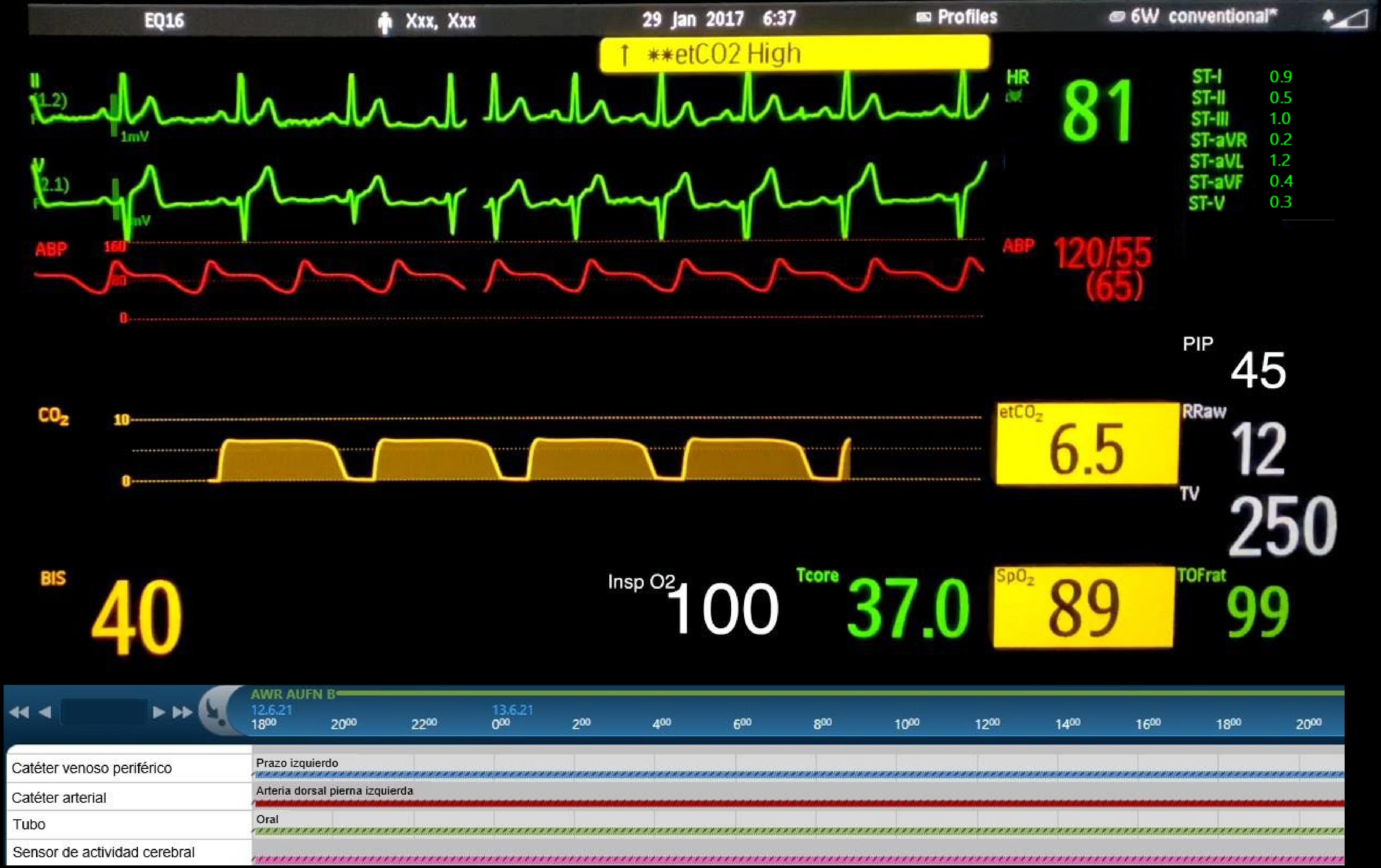
EQ16
Xxx, Xxx
29 Jan 2017 6:37
Profiles
6W conventional*
**etCO2 High
II
1mV
V
ABP
160
80
0
HR
81
ST-I 0.9
ST-II 0.5
ST-III 1.0
ST-aVR 0.2
ST-aVL 1.2
ST-aVF 0.4
ST-V 0.3
ABP
120/55
(65)
PIP
45
CO2
10
0
etCO2
6.5
RRaw
12
TV
250
BIS
40
Insp O2
100
Tcore
37.0
SpO2
89
TOFrat
99
AWR AUFN B
12.6.21
13.6.21
18:00 20:00 22:00 0:00 2:00 4:00 6:00 8:00 10:00 12:00 14:00 16:00 18:00 20:00
Catéter venoso periférico
Prazo izquierdo
Catéter arterial
Arteria dorsal pierna izquierda
Tubo
Oral
Sensor de actividad cerebral

C 5

Thank you so much for your help!
